# Molecular Effects of the CTG Repeats in Mutant Dystrophia Myotonica Protein Kinase Gene

**DOI:** 10.2174/138920208786847944

**Published:** 2008-12

**Authors:** Beatriz Llamusí, Ruben Artero

**Affiliations:** Department of Genetics, University of Valencia, Doctor Moliner, 50, E46100 Burjasot, Valencia, Spain

## Abstract

Myotonic Dystrophy type 1 (DM1) is a multi-system disorder characterized by muscle wasting, myotonia, cardiac conduction defects, cataracts, and neuropsychological dysfunction. DM1 is caused by expansion of a CTG repeat in the 3´untranslated region (UTR) of the *Dystrophia Myotonica Protein Kinase* (*DMPK*) gene. A body of work demonstrates that *DMPK* mRNAs containing abnormally expanded CUG repeats are toxic to several cell types. A core mechanism underlying symptoms of DM1 is that mutant *DMPK* RNA interferes with the developmentally regulated alternative splicing of defined pre-mRNAs. Expanded CUG repeats fold into ds(CUG) hairpins that sequester nuclear proteins including human Muscleblind-like (MBNL) and hnRNP H alternative splicing factors. DM1 cells activate CELF family member CUG-BP1 protein through hyperphosphorylation and stabilization in the cell nucleus. CUG-BP1 and MBNL1 proteins act antagonistically in exon selection in several pre-mRNA transcripts, thus MBNL1 sequestration and increase in nuclear activity of CUG-BP1 both act synergistically to missplice defined transcripts. Mutant *DMPK*-mediated effect on subcellular localization, and defective phosphorylation of cytoplasmic CUG-BP1, have additionally been linked to defective translation of p21 and MEF2A in DM1, possibly explaining delayed differentiation of DM1 muscle cells. Mutant *DMPK* transcripts bind and sequester transcription factors such as Specificity protein 1 leading to reduced transcription of selected genes. Recently, transcripts containing long hairpin structures of CUG repeats have been shown to be a Dicer ribonuclease target and Dicer-induced downregulation of the mutant *DMPK* transcripts triggers silencing effects on RNAs containing long complementary repeats. In summary, mutant *DMPK* transcripts alter gene transcription, alternative splicing, and translation of specific gene transcripts, and have the ability to trigger gene-specific silencing effects in DM1 cells. Therapies aimed at reversing these gene expression alterations should prove effective ways to treat DM1.

## INTRODUCTION

Myotonic Dystrophy type 1 (DM1; OMIM 160900) is a particularly complex inherited disease both from the clinical and the genetic standpoints. Clinically, it affects up to 11 organ systems including the muscular, nervous, ocular, digestive, respiratory and cardiovascular systems. Characteristic disabilities are loss of muscle strength, with a distal to proximal pattern, myotonia, excessive daytime sleepiness, excessive fatigue, abdominal pain as well as dysphagia [1, 2]. Although rare, it is estimated that the worldwide prevalence ranges between 2.1 and 14.3 per 100,000 [3, 4], being the most common form of muscular dystrophy in adults. Genetically, DM1 involves a CTG trinucleotide repeat expansion in the 3´ untranslated region of the *Dystrophia Myotonica Protein Kinase *(*DMPK*) gene, which is assigned to chromosome band 19q13.2-q13.3 [5-9]. DM1 was the first dominantly inherited disease found to be caused by non-coding repeat expansions; the mutation is transcribed into RNA but not translated into protein. Soon after, a second mutation in chromosome 3 was identified in patients with very similar symptoms that were classified as either Proximal Myotonic Myopathy (PROMM) or Myotonic Dystrophy type 2 (DM2) [10, 11]. DM2 was found associated with CCTG tetranucleotide repeat expansions in the first intron of the *Zinc Finger Protein 9 (ZNF9*) gene ([10, 12] reviewed in [13]). Thus, DM1 and DM2 originate from long non-coding repeat expansions, which cause a similar chronic, slowly progressing, multisystemic disease with a dominant inheritance pattern. If the proteins encoded by *DMPK* and *ZNF9* were normal, what was the molecular basis of the dominant phenotype?

In the last few years, a growing body of research, has resulted in a better understanding of the DM1 pathogenesis. Several excellent recent reviews appropriately update the reader to the current state of this knowledge [14]. In this present review, we will specifically focus on the levels of gene expression regulation that mutant *DMPK* affects, namely gene transcription, alternative splicing, gene silencing and RNA translation, with particular emphasis on recent data.

## GENE TRANSCRIPTION CHANGES IN DM1: CIS-EFFECTS

Most dominant disorders are caused by the altered function of a mutant protein product so it was very difficult to explain how a mutation in a non-coding region could cause the multisystemic features of DM1. Since the discovery of the genetic mutation responsible for DM1, much effort has been devoted to precisely define whether *DMPK* transcripts and/or protein levels change in the presence of CTG expansions. Initial reports using antisera and semiquantitative reverse transcriptase-polymerase chain reaction reported decreased levels of both *DMPK* mRNA and protein in adult forms of DM1, suggesting that large CTG repeat expansions altered *DMPK* mRNA synthesis and processing [15-18]. These observations and the detection of mutant *DMPK* transcripts retained in the nucleus of DM fibroblasts and muscle biopsies supported the hypothesis of loss of *DMPK* function (*DMPK* haploinsufficiency) as the mechanism of pathogenesis of DM1 [19, 20]. However, subsequent improvements of protocols used for detection and quantification of total cytoplasmic mRNA levels reported that there was no significant change in *DMPK* transcription [21], and discovered that the anti-DMPK antisera used in previous reports crossreacted with other proteins [22]. Posterior studies confirmed that *DMPK* transcription was altered in patients and that there was no correlation between CTG repeat length and DMPK protein reduction [23-25].

The functional implications of a reduction in DMPK expression were genetically tested with the generation of knockout mice. *DMPK*^-/-^ mice develop a mild late-onset, progressive skeletal myopathy which suggested that *DMPK* might be necessary for the maintenance of skeletal muscle structure [26]. Subsequent studies showed that *DMPK*-deficient mice also exhibited some cardiac conduction abnormalities [27] and metabolic impairment such as abnormal glucose tolerance, reduced glucose uptake and impaired insulin-dependent GLUT4 trafficking in muscle [28]. As similarly proposed for mutant *DMPK*, recent studies also suggest that haploinsufficiency of *ZNF9* may contribute to the DM2 disorder. *ZNF9* knockout mice show phenotypes that resemble DM, including muscle histopathology, myotonia and heart conduction abnormalities [29]. However, the fact that *DMPK*^-/-^ mice showed only a mild phenotype for just some of the DM symptoms and that no *DMPK* point mutations have been associated with a DM1 phenotype strongly suggested that the multisystemic features of DM1 were not simply caused by *DMPK* haploinsufficiency.

**Fig. (1):**
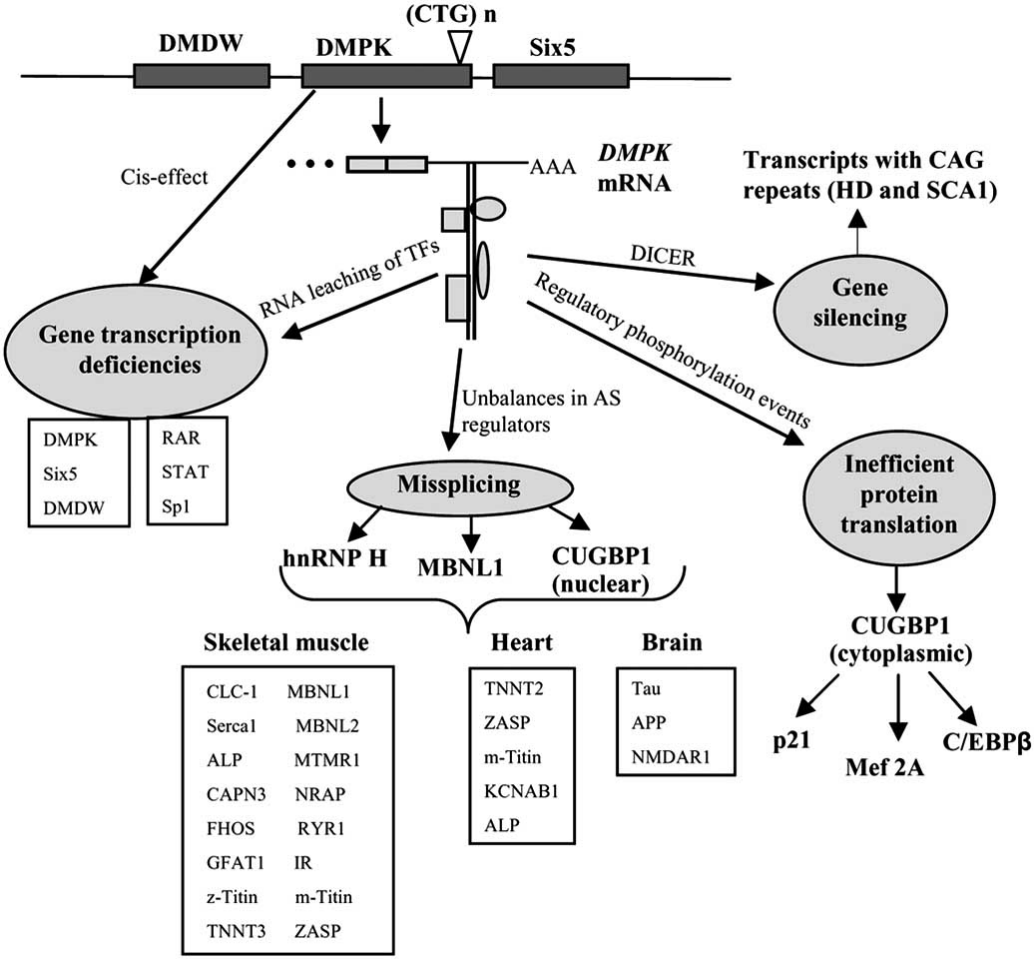
**CTG trinucleotide expansions interfere with gene expression regulation at several levels**. The genomic organization around CTG expansions is represented at the top of the figure, with boxes denoting genes and a line representing the intergenic DNA. CTG-induced local changes in chromatin organization may explain reduced transcription in *DMPK, Six5* and *DMDW* genes (“Cis-effect”). Upon transcription, non-coding CTG expansions fold themselves into dsRNA hairpins in the cell nucleus that aberrantly bind different types of nuclear factors (box, rectangle, small oval and circle). Nuclear retention of mutant *DMPK* transcripts and activity of Dicer ribonuclease may also contribute to the reduction in *DMPK* expression. Sequestration of transcription factors (“RNA leaching of TFs”), and in particular Sp1, has been shown to reduce transcription originating from the *CLCN1* promoter. Aberrant binding of MBNL1 to the ds(CUG) hairpins sequesters the protein. Steady-state levels of nuclear CUG-BP1 increase by protein kinase C mediated phosphorylation. Both effects result in unbalanced cytoplasmic levels of these antagonic alternative splicing (AS) regulators leading to changes in the transcript isoforms originating from several pre-mRNAs (examples listed in the boxes under the “Skeletal muscle”, “Heart” and “Brain” labels). A combination of nuclear stabilization and changes in key phosphorylation events likely explain the inefficient activation of *p21, Mef2A* and *C/EBPβ* mRNA translation by cytoplasmic CUG-BP1. ds(CUG) hairpins are a substrate for the Dicer ribonuclease, which reduces levels of mutant *DMPK* simultaneously generating CUG siRNA with the ability to silence transcripts containing complementary CAG repeats such as those of the Huntington Disease and Spinocerebellar ataxia genes. The figure includes both human and mouse genes, the expression of which is known to be altered in DM1 or animal models of the disease.

The possibility that CTG and CCTG repeat expansions were altering the local structure of the chromatin (a cis-effect; Fig. **1**), thus interfering with the expression of *DMPK* and neighbouring genes, has also been considered. Immediately adjacent to *DMPK* are *SIX5*, which encodes a homeodomain transcription factor, and *myotonic dystrophy gene with WD repeats* (*DMDW*) that is prominently expressed in testis and brain [30-32]. The hypothesis is chiefly supported by the fact that *DMDW* expression levels are reported decreased in repeat expansion bearing patients [31, 33]. Moreover, mutant analysis in *Drosophila *has shown that *D-Six4*, the closest *Six5* homolog in flies, is required for the normal development of muscle and the mesodermal components of the gonad. This suggested that human Six5 could participate in muscle wasting and testicular atrophy phenotypes in DM1 [34]. However, *Six5* knockout mice only develop cataracts that lack the distinctive iridescent opacities characteristic of cataracts from DM1 patients [35].

## GENE TRANSCRIPTION CHANGES IN DM1: TRANS-EFFECTS

Electron microscope examination has revealed that CUG repeat RNA forms double-stranded RNA (dsRNA) segments [36] that, additionally, are able to bind the dsRNA activated protein kinase PKR [37] and Muscleblind protein family members [38]. More recent crystallographic data have confirmed that CUG repeat RNA forms antiparallel double-stranded helices similar in structure to A-form RNA except for the unique U-U-mismatches [39]. This peculiar structure supported an RNA dominant mutation model in which long dsRNAs gain the capacity to sequester certain RNA-binding proteins that are correspondingly depleted from their normal subcellular localizations.

A number of regulatory transcription factors (TFs) have been found to change their nuclear compartment distribution from active chromatin to CUG repeat-containing ribonuclear particles in a DM1 cell model, a process known as RNA leaching [40]. Measured fractions of Specificity protein 1 (Sp1), Signal Transducer and Activator of Transcription (STAT1 and STAT3) and Retinoic Acid Receptor gamma (RARγ) in the chromatin, were dramatically reduced after three weeks of mutant *DMPK* RNA expression. Diverse genes are consequently reduced in expression, including the muscle-specific *chlorine channel 1 (CLCN1)*, which has been involved in myotonia [40]. Another example of TF found to be altered by repeat expansions is MyoD. Levels of MyoD, a TF necessary for muscle differentiation and regeneration, are significantly reduced in myoblasts expressing the mutant 3'-UTR *DMPK* RNA [41]. Changes in TF activity should alter transcription of target genes (a trans-effect; Fig. **1**). Consistently, gene transcription changes in DM has been further verified by comparing the expression profile of muscle biopsies from DM1 and DM2 patients to controls in a macroarray analysis of 96 neuroscience-related genes [42]. Six genes critical for calcium and potassium metabolism and mitochondrial functions were misregulated both in DM1 and DM2 also strengthening the current view that both diseases share a common pathogenetic pathway [42].

DM1 has been also associated with overexpression of the cardiac-specific transcription factor *NK2 transcription factor related, locus 5 (Drosophila) (Nkx2-5)*, also known in *Drosophila* as *tinman*. Levels of NKX2-5 increased in cardiac tissue in a reversible transgenic mouse model for RNA toxicity [43]. Moreover, overexpression of *DMPK* 3' UTR mRNA in mouse skeletal muscle also induced transcriptional activation of Nkx2-5 and its targets. In human muscles these changes were specific to DM1 and were not present in other muscular dystrophies possibly contributing to the cardiac conduction degeneration found in DM [43].

Despite still limited experimental support, modification of TFs expression by leaching from chromatin or other mechanisms provides a powerful explanation for broad gene expression changes, especially if their effects add on alternative splicing changes already known to occur in DM1 (see below). A requirement for general transcription factors in different tissues that express *DMPK* would account for the multisystemic and multisymptomatic nature of DM1. Moreover, a common trans-acting mechanism with trapping and depletion of similar TFs might contribute to the clinical analogies between DM1 and DM2. 

## CUG REPEAT RNA SEQUESTERS ALTERNATIVE SPLICING FACTORS

The identification of RNA-binding proteins bound to CUG repeat expansions clearly suggested an RNA-gain-of function model for DM1 [38] and provided a molecular explanation for the phenotypical similarities between DM1 and DM2 since the same, or similar RNA-binding proteins, could get sequestered by mutant CUG and CCUG repeat RNA. Several studies have established that one of the reasons for the toxicity of mutant *DMPK* RNA is that it interferes with the activity of human Muscleblind-like 1, 2 and 3 (MBNL1-3) proteins. MBNL proteins were directly implicated in DM1 pathogenesis when they were found to specifically bind 20 CUG trinucleotide repeats or longer *in vitro* in HeLa cell extracts [38]. MBNL proteins are orthologous to the *Drosophila* Muscleblind proteins, which are required for terminal differentiation of muscle and photoreceptor cells [44, 45]. Co-localization studies in both cell and tissue samples from DM1 and DM2 patients showed that MBNL1-3 were retained in the cell nucleus as ribonuclear foci that included mutant transcripts [46-52]. *Drosophila* Muscleblind similarly co-localized with CUG ribonuclear foci in muscle cells in DM1 fly models [53-55]. Human MBNL proteins seem to have acquired specialized functions although all three were capable of regulating the use of alternative exons in *cardiac troponin T (cTNT)* and *insulin receptor (IR*) transcripts [56]. While MBNL1 is involved in promoting muscle differentiation, MBNL3 represses it and MBNL2 participates in the subcellular localization of *alpha3-integrin* transcripts [38, 57, 58]. Extensive alternative splicing in the MBNL1, 2 and 3 genes generates at least nine, three and six protein isoforms, respectively [59].

Consistent with the involvement of MBNL proteins in the RNA gain of function mechanism, *Mbnl1* knockdown mice [60] showed DM1-like phenotypes including iridescent cataracts, myotonia and missplicing of muscle transcripts that had been reported altered in DM1 patients such as *IR* and *CLC-1* pre-mRNA [61, 62]. *muscleblind* mutant embryos similarly showed altered expression of muscle transcript isoforms from the *Drosophila ZASP* homologue and *alpha-actinin* genes [63]. More recently, the generation of a *Mbnl2*-deficient mouse also displaying myotonia, skeletal muscle pathology consistent with human DM, and reduced expression of *CLC-1* mRNA in skeletal muscle, suggests that depletion of MBNL2 might also contribute to the human DM pathogenesis [64]. Conversely, overexpression of MBNL1 *in vivo* using a recombinant adeno-associated viral vector rescued disease-associated muscle myotonia and adult-splicing patterns of muscle pre-mRNAs characteristically misspliced in transgenic mice expressing 250 CTG repeats in the 3´UTR of a human skeletal *alpha-actin* transcript. These results support the hypothesis that loss of MBNL1 activity is a primary pathogenic event in the development of the disease [65].

CUG repeat expansions interfere with the activity of alternative splicing factors other than Muscleblind. CUG-BP1 and ETR-3 like factor (CELF) member CUG-BP1 does not bind ds(CUG) hairpins nor co-localizes with ribonuclear foci but its activity is increased in DM1 myoblasts, skeletal muscle, and heart tissues [36, 66, 67] (see [68] for a review about CELF RNA binding proteins). Although the molecular mechanism leading to CUG-BP1 activation is not completely understood, inappropriate activation of the protein kinase C (PKC) pathway contributes to the pathogenic effect of noncoding CUG repeat RNA through hyperphosphorylation of nuclear CUG-BP1, which is stabilized in this cellular compartment [69]. Transgenic mice that overexpress CUG-BP1 in muscle and heart reproduce missplicing alterations typical of DM1, which has confirmed the involvement of this protein in the pathogenesis of the disease as well as the antagonism between MBNL1 and CUG-BP1 in alternative splicing regulation ([70] and below).

Results from several groups support that increased activity of CUG-BP1 in DM1 is pathogenic as CUG-BP1 regulates alternative splicing of pre-mRNA transcripts antagonizing Muscleblind activity [67, 71-73]. Another splicing regulator altered by CUG repeat expansions is heterogeneous nuclear ribonucleoprotein H (hnRNP H). The protein was identified in UV-crosslinking assays as a factor capable of binding and modulating nuclear retention of mutant *DMPK* mRNA. The specific binding of hnRNP H requires not only a CUG expansion but also a splicing branch point distal to the repeats [73]. It has been proposed that in normal myoblast hnRNP H and CUG-BP1 form an RNA-dependent complex required to maximally inhibit *IR* exon 11 inclusion, which is counteracted by the exon 11 splice enhancing activity of Muscleblind proteins [74]. Because Muscleblind proteins are required facilitators of *IR* exon 11, exclusion of exon 11 in *IR* transcripts is a typical splicing misregulation event in DM1 [61].

Given the involvement of alternative splicing regulators Muscleblind, CUG-BP1 and hnRNP H, it is not surprising that a defining molecular alteration in DM1 is missplicing of a defined set of muscle, brain and heart transcripts (for a compilation of splice alterations in DM1 see [75]). Changes in tissue-specific splice patterns have been shown to contribute to particular symptoms in DM1 patients. Myotonia has been attributed to reduced expression of *Clcn1* due to the combined effects of a decrease in transcription from the *Clcn1* gene and inclusion of alternative exon 7a, which includes a TGA stop codon that triggers non-sense mediated decay [76]. Indeed, patch clamp analysis has confirmed that myotonia is associated with a marked reduction in chloride channel activity [77] thus suggesting that reduction in transcription and generation of protein variants with no intrinsic channel activity originate myotonia [50, 77, 78]. The number of genes and alternative exons altered in DM1 is still unknown, but the data available support a view in which the process of constitutive RNA processing is not affected in DM1 cells [56, 79, 80]. During embryonic development and postnatal periods in mammals, a series of changes are required at the transcriptional and post-transcriptional levels that account for the remodeling necessary for the adult body development [81]. Some of these changes are based on the alternative splicing of many transcripts in these tissues, which are regulated by RNA binding proteins including members of the Muscleblind and CELF families. Activation of MBNL1 during muscle differentiation stages brings about the exclusion of fetal exons from multiple RNA transcripts leading to their adult encoding isoforms [82, 83]. Indeed, the DM pathology seems to selectively affect a defined set of pre-mRNAs that undergo such developmentally regulated switch in their alternative splicing pattern. Failure to switch on an adult-type alternative splicing pattern, due to lack of Muscleblind function, results in the maintenance of a fetal-like splicing pattern in adult tissue [82, 83]. A trans-effect on the alternative splicing of many RNAs that leads to the expression of splice products developmentally inappropriate for a particular tissue has been recently termed spliceopathy [75] being DM1 the first example of spliceopathy in humans (Fig. **1**).

Although the notion of MBNL1 sequestration by CUG repeats is certainly the best established, a number of results suggest that the toxic effect caused by CUG-repeat expansion RNAs might involve more than just Muscleblind sequestration. First, disruption of MBNL1-regulated splicing and formation of RNA foci are separable events in cell culture conditions. Both CUG and CAG repeat expansions form ribonuclear foci that colocalize with MBNL1 in COSM6 cells, but only CUG repeats disrupt MBNL1-regulated splicing [84]. Second, MBNL1 bound 70 CAG repeats in a yeast three-hybrid assay [85] whereas 162 CUG repeats readily sequestered Muscleblind but failed to induce any discernible phenotype in a *Drosophila* DM1 model [53]. Finally, it has been recently shown that non-coding CAG repeat RNA of pathogenic length induced progressive neural dysfunction in *Drosophila* but, as similarly reported by Ho *et al* 2005, 270 CAG repeats did not change the profile of alternative splicing of a reporter construct [84, 86].

Among genetic diseases the therapeutic opportunities in DM1 are particularly favourable because elimination of toxic RNA reversed cardinal features of DM1 in a transgenic mice model. Overexpression of a normal *DMPK* 3´UTR (five CUG repeats) as part of an inducible RNA transcript was reported to cause myotonia, cardiac conduction abnormalities, histopathology and RNA splicing defects in the absence of detectable nuclear inclusions. Five CUG repeats were apparently sufficient to originate DM1-like phenotypes without formation of RNA or Mbnl nuclear foci. In these transgenic mice CUG-BP1 levels were found increased in skeletal muscle while no change in Mbnl1 was detected. Although surprising, the authors explained the DM1 phenotype by the unbalance between CUG-BP1 (upregulated) and Mbnl1 (unchanged) antagonistic activities as alternative splicing regulators resulting in missplicing of characteristic transcripts such as *ClC-1* and *cTNT* [72]. The mapping of MBNL1-binding sites upstream of their normal splicing targets, however, offers an alternative view. RNA recognition by MBNL proteins involves a common mechanism for both normal targets and pathogenic repeats, which implies recognition of GC-rich hairpins containing pyrimidine mismatches [87]. Results from this work suggest that below a certain length threshold (<20 repeats), the A-helix structure formed by dsCUG [39] is unstable and that ssCUG are not appropriate binding targets for MBNL1. It seems that MBNL1 recognizes relatively short GC-rich hairpins (18 nucleotides) only if the overall RNA secondary structure is stabilized by additional sequence interactions [87]. Thus, overexpression of CUG repeats within the normal range, such as for example in the inducible model reported by the Mahadevan´s group [72], might readily divert MBNL1 from their normal targets in the absence of long CUG expansions.

## GENE SILENCING: dsCUG EXPANSIONS ARE DICER TARGETS

The double stranded nature of CUG repeat expansions *In vivo* prompted speculations as for the possibility that might act as a Dicer ribonuclease target thus generating small interfering RNAs (siRNAs) that would silence transcripts containing complementary, or near complementary sequences (Fig. 1) [88]. Suggestively, MBNL1 protein itself includes a run of seven alanines encoded by a sequence with the potential to act as target of CUG siRNAs. In vitro studies in HeLa cells have already shown that the mechanism that permits the specific silencing of target RNAs by siRNA and miRNA is active in the cell nucleus [89]. Indeed, Krol and co-workers [90] have shown that transcripts containing long hairpin structures composed of CNG repeats are a class of Dicer ribonuclease targets. Dicer activity elicits two opposing effects in DM fibroblast cells [90]. On the one hand, the activity of the ribonuclease downregulates the mutant DMPK transcript, which removes a toxic RNA from the cell and may contribute to the reduction of DMPK expression levels. On the other hand, the Dicer-induced short CUG repeats generated act as siRNAs and use the RNA interference pathway to silence expression of transcripts containing long complementary repeats, which may add up to potentially pathogenic gene expression unbalances.

## EFFECTS ON mRNA TRANSLATION

CUG-BP1 is a multifunctional RNA-binding protein that regulates RNA processing at several stages including activating cap-dependent and cap-independent translation, RNA stability and splicing [83, 91]. Although it was already known that cytoplasmic CUG-BP1 could promote translation of *p21* [92], *C/EBPβ* [93] and *Mef2A* [70] in differentiating cells by interacting with translation initiation factor eIF2, the mechanism by which translation was inefficient in DM1 muscle cells was not fully understood, in particular after confirming that the levels of CUG-BP1 mRNA did not show any significant change in DM1 muscle cells when compared to normal myoblasts [94]. In a recent paper Salisbury and coworkers [91] showed that two key phosphorylation events in CUG-BP1 were mis-regulated during DM1 myogenesis. Serine/threonine-specific protein kinase family member Akt phosphorylated CUG-BP1 at Ser28 and increased interaction of CUG-BP1 with cyclin D1 mRNA, which is a strong promoter of cell proliferation. CUG-BP1 was also phosphorylated at Ser302 by cyclinD3/cdk4 complexes. Phosphorylation of CUG-BP1 by cyclinD3/cdk4 increased interactions of CUG-BP1 with C/EBPbeta and p21 mRNAs. Cyclin D3-cdk4-mediated phosphorylation of CUG-BP1 increased formation of the translational CUG-BP1-eIF2 complexes during normal muscle differentiation. Because examination of DM1 cells revealed that both cyclin D3 and cdk4 levels did not increase in DM1 differentiating cells, reduction in formation of the translational complex CUG-BP1-eIF2 provides a reasonable explanation for the reduction in p21 and Mef2A translation in DM1 myoblasts. Confirmation that cyclinD3/cdk4 was implicated in CUG-BP1 translational control came from experiments in which ectopic expression of cyclin D3 corrected the differentiation of DM1 myocytes [91]. Failure to express cell cycle arrest control p21 protein, as well as others with similar function, correlates well with defective withdraw from cell cycle of cultured DM muscle cells, a necessary step for muscle differentiation [66]. Furthermore, whereas differentiated normal myoblasts accumulated CUGBP1 in the cytoplasm, skeletal muscle cells from DM1 patients failed to induce cytoplasmic levels of a CUG RNA binding protein, also contributing to the inhibition of translation of key muscle and cell cycle transcripts [66].

## PERSPECTIVES

The toxic RNA hypothesis is now a well established model to explain the pathogenic effect of mutant *DMPK* transcripts and is being extended to other trinucleotide diseases such as Fragile X Tremor Ataxia Syndrome, Spinocerebelar Ataxia 3 (SCA3), or Huntington´s Disease-Like 2 (HDL-2) among other disorders [86, 95, 96]. Despite the fact that it builds on results obtained over more than a decade of work, a number of key issues still deserve immediate attention. First, low through-put technologies applied so far provide a low resolution picture of the complex pattern of gene transcription changes taking place in the disease state. Use of microarray and/or ultrasequencing technologies should provide an accurate genome-wide description of gene changes both at the transcription and pre-mRNA maturation levels, with the possibility of identifying new, unexpected, additional levels of complexity in the disease. Second, recent results indicate that key signal transduction components such as protein kinase C and Akt become altered in DM1 cells [69, 91]. Whether this is a primary or secondary effect of CUG repeat RNA remains to be determined as well as the relevance of signal transduction changes to the regulation of activity of alternative splicing factors themselves. Finally, the generation of improved animal models for Myotonic Dystrophy should allow for the development and evaluation of effective therapies. In the last few years DM models in *Drosophila*, mice and *C.elegans* have been generated and tested for several potential treatments ranging from activation of the RNA interference pathway to the administration of chemical compounds [55, 72, 97-99]. Development of and effective anti-DM1 treatment will require to further explore these and newer experimental therapies in animal models, as well as keep untangling the still unexplored complexity of this multifaceted disease.
